# BET bromodomain inhibitors and agonists of the beta-2 adrenergic receptor identified in screens for compounds that inhibit DUX4 expression in FSHD muscle cells

**DOI:** 10.1186/s13395-017-0134-x

**Published:** 2017-09-04

**Authors:** Amy E. Campbell, Jonathan Oliva, Matthew P. Yates, Jun Wen Zhong, Sean C. Shadle, Lauren Snider, Nikita Singh, Shannon Tai, Yosuke Hiramuki, Rabi Tawil, Silvère M. van der Maarel, Stephen J. Tapscott, Francis M. Sverdrup

**Affiliations:** 1 Human Biology Division, Fred Hutchinson Cancer Research Center, Seattle, WA 98109 USA; 2 Edward A. Doisy Department of Biochemistry and Molecular Biology, Saint Louis University, Saint Louis, MO 63104 USA; 3 Molecular and Cellular Biology Program, University of Washington, Seattle, WA 98105 USA; 4 Department of Neurology, University of Rochester Medical Center, Rochester, NY 14642 USA; 5 Department of Human Genetics, Leiden University Medical Center, 2333 ZA Leiden, The Netherlands; 6 Department of Neurology, University of Washington, Seattle, WA 98105 USA

**Keywords:** Facioscapulohumeral muscular dystrophy, FSHD, DUX4, Bromodomain, Adrenergic, High-throughput screening

## Abstract

**Background:**

Facioscapulohumeral dystrophy (FSHD) is a progressive muscle disease caused by mutations that lead to epigenetic derepression and inappropriate transcription of the double homeobox 4 (*DUX4*) gene in skeletal muscle. Drugs that enhance the repression of *DUX4* and prevent its expression in skeletal muscle cells therefore represent candidate therapies for FSHD.

**Methods:**

We screened an aggregated chemical library enriched for compounds with epigenetic activities and the Pharmakon 1600 library composed of compounds that have reached clinical testing to identify molecules that decrease *DUX4* expression as monitored by the levels of DUX4 target genes in FSHD patient-derived skeletal muscle cell cultures.

**Results:**

Our screens identified several classes of molecules that include inhibitors of the bromodomain and extra-terminal (BET) family of proteins and agonists of the beta-2 adrenergic receptor. Further studies showed that compounds from these two classes suppress the expression of DUX4 messenger RNA (mRNA) by blocking the activity of bromodomain-containing protein 4 (BRD4) or by increasing cyclic adenosine monophosphate (cAMP) levels, respectively.

**Conclusions:**

These data uncover pathways involved in the regulation of *DUX4* expression in somatic cells, provide potential candidate classes of compounds for FSHD therapeutic development, and create an important opportunity for mechanistic studies that may uncover additional therapeutic targets.

**Electronic supplementary material:**

The online version of this article (doi:10.1186/s13395-017-0134-x) contains supplementary material, which is available to authorized users.

## Background

Facioscapulohumeral dystrophy (FSHD) is a prevalent muscular dystrophy affecting over 800,000 individuals worldwide. The disease typically presents in young adults as facial and upper extremity weakness, and progresses to involve nearly all skeletal muscle groups [1]. FSHD is caused by the mis-expression of the double homeobox 4 (DUX4) transcription factor in skeletal muscle. *DUX4* is encoded by a retrogene located in each unit of the D4Z4 macrosatellite repeat array in the subtelomeric region of chromosomes 4q and 10q, and is normally expressed in the pre-implantation embryo and in germline tissues where it activates early developmental and stem cell genes [1–4]. In most somatic tissues, including skeletal muscle, the D4Z4 arrays and *DUX4* are epigenetically silenced through multiple mechanisms that suppress repetitive elements in the genome [5–9].

FSHD results from a contraction at 4q35 resulting in too few D4Z4 repeats for efficient repeat-mediated epigenetic repression (FSHD type 1, FSHD1) or from the presence of mutations in trans-acting chromatin factors necessary for epigenetic repression of the D4Z4 array (FSHD type 2, FSHD2) [10–12]. Inefficient D4Z4 repression, when combined with a permissive chromosome 4qA haplotype that provides a polyadenylation site for the DUX4 messenger RNA (mRNA), results in the ectopic expression of DUX4 protein in muscle cells [1, 5, 10]. DUX4 mis-expression in skeletal muscle induces early embryo, stem cell, and germline genes; activates repetitive elements; suppresses innate immune response and nonsense-mediated RNA decay pathways; inhibits myogenesis; and causes cell death through mechanisms that include the accumulation of aberrant and double-stranded RNAs [13–22].

Because of its causative role in FSHD, suppressing *DUX4* expression is a primary therapeutic approach for halting disease progression. However, the mechanisms responsible for *DUX4* expression are poorly understood and limited drug targets have been identified. Consequently, there is currently no treatment available for FSHD and few clinical trials of promising therapies are ongoing.

Here, we screened an aggregated chemical library enriched for compounds with epigenetic activities and the Pharmakon 1600 library composed of compounds that have reached clinical testing to identify molecules that decrease *DUX4* expression as monitored by the levels of DUX4 target genes in FSHD patient-derived muscle cells. Our screens identified bromodomain and extra-terminal (BET) bromodomain inhibitors and beta-2 adrenergic receptor agonists as classes of compounds that suppress *DUX4* expression. These findings illuminate pathways that regulate *DUX4* expression in somatic cells and provide initial candidate molecules for FSHD therapeutic development.

## Methods

### Compounds

The Pharmakon 1600 drug library was obtained from MicroSource Discovery Systems, Inc. (Gaylordsville, CT, USA). The collection of epigenetic modulator compounds was composed of the Epigenetics Screening Library from Cayman Chemical (Ann Arbor, MI, USA), the Epigenetics Compound Library from Selleck Chemicals LLC (Houston, TX, USA), and novel epigenetic probes acquired from the Structural Genomics Consortium (www.thesgc.org). Screening compounds were delivered in microplates as 10 mM stocks dissolved in dimethyl sulfoxide (DMSO) and kept at −80 °C until use. Individual compounds used in follow-up testing were purchased from Sigma-Aldrich (St. Louis, MO, USA), Tocris Bioscience (Bristol, UK), or Selleck Chemicals, dissolved in DMSO at a 10 mM stock concentration and stored at −80 °C.

### Cell culture

Primary human myoblast cell lines were obtained from the Fields Center at the University of Rochester (https://www.urmc.rochester.edu/neurology/fields-center.aspx) and immortalized by retroviral transduction of cyclin-dependent kinase 4 (CDK4) and human telomerase reverse transcriptase (hTERT) [23]. Immortalized myoblasts were grown in Ham’s F-10 Nutrient Mix (Gibco, Waltham, MA, USA) supplemented with 20% HyClone Fetal Bovine Serum (GE Healthcare Life Sciences, Pittsburgh, PA, USA), 100 U/100 μg penicillin/streptomycin (Gibco), 10 ng/ml recombinant human fibroblast growth factor (Promega Corporation, Madison, WI, USA), and 1 μM dexamethasone (Sigma-Aldrich). Differentiation of myoblasts into myotubes was achieved by switching the fully confluent myoblast monolayer into Dulbecco’s Modified Eagle Medium (DMEM) (Gibco) containing 1% horse serum (Gibco), 100 U/100 μg penicillin/streptomycin, 10 μg/ml insulin (Sigma-Aldrich), and 10 μg/ml transferrin (Sigma-Aldrich) (HS/IT media) or DMEM/Nutrient Mixture F-12 (1:1, Gibco) supplemented with 2% KnockOut Serum Replacement (Gibco), 100 U/100 μg penicillin/streptomycin, 10 μg/ml insulin, and 10 μg/ml transferrin (KSR media) for 2–6 days. The details of each cell line used in this study are provided in Additional file 1: Table S1.

### Epigenetic modifier library screen

Transient DNA transfections of 54-2 FSHD1 myoblasts were performed using TransIT-2020 (Mirus Bio LLC, Madison, WI, USA) according to the manufacturer’s specifications. Briefly, 15 × 10^6^ cells were suspended in 25 ml growth media on the day of transfection. Cells were co-transfected with 16.2 μg of a pGL3-basic reporter vector carrying the intact or mutated zinc finger and SCAN domain-containing 4 (*ZSCAN4*) promoter fragment upstream of the firefly luciferase gene [13] and 1.8 μg of a pRL-CMV-*Renilla* luciferase control reporter vector. DNAs were diluted in 1.8 ml Opti-MEM Reduced Serum Medium (Thermo Fisher Scientific, Waltham, MA, USA) with 45 μl TransIT-2020 transfection reagent. Suspended cells were combined with the transfection mixture and plated in a T175 flask. The next day, cells were trypsinized and re-plated at 5 × 10^4^ cells/well in 96-well plates. Growth media was removed and replaced with HS/IT differentiation media the following day. Compounds were added the next day and cells were allowed to differentiate for an additional 96 h. Luminescence was quantified using the Dual-Luciferase Reporter Assay System (Promega) and a SpectraMax L microplate luminometer (Molecular Devices LLC, Sunnyvale, CA, USA) following the manufacturers’ directions.

### Pharmakon 1600 library screen

Compounds were diluted 1:2000 from DMSO stocks into KSR differentiation media so that DMSO was limited to 0.05%, and the initial screening concentration was 5 μM with one well per compound. Media containing compounds was added to confluent monolayers of MB200 FSHD2 myoblasts in 96-well plates. Forty-two hours later, each well was visually inspected and documented before cells were harvested for mRNA expression analysis. Each 96-well screening plate contained 8 positive (I-BET762, 1 μM) and 8 negative (DMSO) controls such that every row included one of each type of control. The sequential addition of cell lysis reagents, which proceeded by row, resulted in slight variations in the maximal and minimal PCR signals from row to row. Consequently, a marginal Z-prime of 0.217 was calculated from the positive and negative controls for the entire screen (20 plates) with a coefficient of variation of 0.23. To compensate for this variability, data in each row were normalized to the negative control in that row and hits selected as compounds that resulted in > 65% target mRNA inhibition relative to the negative control. A plot of the primary screening data after row by row normalization is provided in Additional file 2: Figure S1.

### DUX4 activity assay

Briefly, 6 × 10^5^ 54-6 control (non-FSHD) myoblasts in 1 well of a 6-well plate were co-transfected with 75 ng of the DUX4 expression vector pCS2-DUX4 [13] and 2.925 μg of pGL3-promoter vector (Promega) using Lipofectamine 3000 (Thermo Fisher Scientific) following the manufacturer’s instructions. Three hours after transfection, cells were trypsinized and distributed to 84 wells of a 96-well plate. Two hours later (a time at which there is a low but detectable level of DUX4 target gene expression), 12 wells were harvested to represent the “baseline” gene expression state while compounds were added to the remaining wells. Nineteen hours later, the remaining wells were harvested to represent the “endpoint” gene expression state. DUX4 activity was determined by comparing DUX4 target gene mRNA levels at the 24 h “endpoint” to the levels at the 5 h “baseline.”

### mRNA expression analyses

For screening of the Pharmakon 1600 library and for the DUX4 activity assay, cell lysates were prepared using Cells-to-Ct Bulk Lysis Reagents (Invitrogen, Waltham, MA, USA). Quantitative polymerase chain reaction (PCR) was carried out on a QuantStudio 5 (Applied Biosystems, Waltham, MA, USA) using TaqMan Gene Expression Assays (Applied Biosystems) and TaqMan Fast Virus 1-Step Master Mix (Invitrogen). The relative expression level of DUX4 target gene methyl-CpG binding domain protein 3 like 2 (*MBD3L2*) was normalized to that of the reference gene ribosomal protein L30 (*RPL30*), which was included in multiplex (two gene) PCR reactions. The pair performed well in terms of amplification efficiency. For other gene expression analyses, total RNA was extracted from whole cells using the RNeasy Mini Kit (Qiagen, Hilden, Germany) according to the manufacturer’s instructions. Isolated RNA was treated with DNase I (Thermo Fisher Scientific), heat inactivated, and reverse transcribed into cDNA using Superscript III (Thermo Fisher Scientific) and oligo(dT) primers (Invitrogen) following the manufacturer’s protocol. Quantitative PCR was carried out on a QuantStudio 7 Flex (Applied Biosystems) using primers specific for each mRNA and iTaq SYBR Green Supermix (Bio-Rad Laboratories, Inc., Hercules, CA, USA), or on a QuantStudio 5 using TaqMan Gene Expression Assays and TaqMan Gene Expression Master Mix (Applied Biosystems). The relative expression levels of target genes were normalized to that of the reference genes ribosomal protein L27 (*RPL27*), ribosomal protein L13a (*RPL13A*), or *RPL30* by using the delta-delta-Ct method [24] after confirming equivalent amplification efficiencies of reference and target molecules.

### DNA methylation analysis

DNA methylation analyses were conducted by EpigenDx, Inc. (Hopkinton, MA, USA). Genomic DNA specimens were isolated using the QIAamp DNA Mini Kit (Qiagen) and shipped on dry ice from Saint Louis University to EpigenDx. For each sample, 500 ng of extracted genomic DNA was bisulfite treated using the EZ DNA Methylation Kit (Zymo Research, Irvine, CA, USA). Bisulfite-treated DNA was purified according to the manufacturer’s protocol and eluted in a final volume of 46 μl. PCR was performed using 1 μl of bisulfite-treated DNA and 0.2 μM of each primer for EpigenDx methylation assays ADS3747 and ADS1454. One primer was biotin-labeled and high-performance liquid chromatography-purified in order to capture the final PCR product using sepharose beads. For pyrosequencing, PCR products were bound to Streptavidin Sepharose High Performance (GE Healthcare Life Sciences), after which the immobilized PCR products were purified, washed, denatured with a 0.2 μM NaOH solution, and rewashed using the Pyrosequencing Vacuum Prep Tool (Qiagen) per the manufacturer’s protocol. Next, 0.5 μM of sequencing primer was annealed to the purified single-stranded PCR products. Ten microliters of the PCR products were sequenced by pyrosequencing on the PSQ96 HS System (Qiagen) following the manufacturer’s instructions. The methylation status of each CpG site was determined individually as an artificial C/T single nucleotide polymorphism using the QCpG software (Qiagen). The methylation level at each CpG site was calculated as the percentage of the methylated alleles divided by the sum of all methylated and unmethylated alleles. The mean methylation level was calculated using methylation levels of all measured CpG sites within the targeted region of each gene. Each experiment included non-CpG cytosines as internal controls to detect incomplete bisulfite conversion of the input DNA. In addition, a series of unmethylated and methylated DNA were included as controls in each PCR. Furthermore, PCR bias testing was performed by mixing unmethylated control DNA with in vitro methylated DNA at different ratios (0%, 5%, 10%, 25%, 50%, 75%, 100%), followed by bisulfite modification, PCR, and pyrosequencing analysis.

### Small interfering RNA transfections

Duplex small interfering RNAs (siRNAs) were obtained from Qiagen (FlexiTube) or Thermo Fisher Scientific (Silencer Select). Transfections of siRNAs into myoblasts were carried out using Lipofectamine RNAiMAX (Invitrogen) or Lipofectamine 2000 (Invitrogen) according to the manufacturer’s instructions. For bromodomain-containing protein 2 (BRD2), bromodomain-containing protein 3 (BRD3), and bromodomain-containing protein 4 (BRD4) siRNAs, 1.5 × 10^5^ cells suspended in 1 ml culture media were mixed with 2 μl Lipofectamine 2000 and 12.5 pmol of either gene-specific siRNAs or a scrambled non-silencing control siRNA diluted in 100 μl Opti-MEM Reduced Serum Medium and plated in 1 well of a 12-well plate. Cells were harvested for RNA analysis 72–96 h later. For adrenoceptor beta 2 (ADRB2) siRNA, a double transfection protocol was followed to ensure efficient depletion of pre-existing proteins. Briefly, cells were seeded at ~ 30% confluence in 6-well plates and transfected ~ 20 h later with 6 μl Lipofectamine RNAiMAX and 25 pmol of either gene-specific siRNAs or a scrambled non-silencing control siRNA diluted in 125 μl Opti-MEM Reduced Serum Medium. Forty-eight hours following this, cells were transfected a second time and harvested for RNA analysis 48–72 h later.

### Western blotting

Reduced and boiled samples were run on NuPage 4–12% precast polyacrylamide gels (Invitrogen) and transferred to polyvinylidene difluoride membrane (Invitrogen). After blocking in 5% milk in PBST, membranes were incubated with appropriate primary antibodies in block solution overnight at 4 °C. Membranes were then incubated with horseradish peroxidase-conjugated secondary antibodies in block solution for 1 h at room temperature, and chemiluminscent substrate (Thermo Fisher Scientific) was used for detection.

### Antibodies

The following antibodies were used: α-Tubulin (DM1A), Sigma-Aldrich T9026; β2-AR (R11E1), Santa Cruz Biotechnology (Dallas, TX, USA) sc-81577, lot#G1117; BRD2, Bethyl Laboratories (Montgomery, TX, USA) A302-582A; BRD3, Bethyl A302-368A; BRD4, Bethyl A301-985A50; GAPDH, Thermo Fisher Scientific TAB1001; Histone H3, Abcam (Cambridge, UK) ab1791; and MBD3L2, Abcam ab107999, lot#GR126890-1; rabbit monoclonal antibody against DUX4 (E14-3) was produced in collaboration with Epitomics (Burlingame, CA, USA) and is described elsewhere [25].

### TaqMan gene expression assay ID numbers

BRD2, Hs01121986_g1; BRD3, Hs00201284_m1; BRD4, Hs04188087_m1; BRDT, Hs00976114_m1; MBD3L2, Hs00544743_m1; MYF5, Hs00929416_g1; MYH2, Hs00430042_m1; MYOD1, Hs00159528_m1; MYOG, Hs01072232_m1; RPL13A, Hs04194366_g1; RPL30, Hs00265497_m1; SMCHD1, Hs00826906_m1; TRIM43, Hs00299174_m1; ZSCAN4, Hs00537549_m1; DUX4, primers GCCGGCCCAGGTACCA and CAGCGAGCTCCCTTGCA with probe 6FAMCAGTGCGCACCCCGMGBNFQ.

### Oligonucleotide sequences

#### Quantitative reverse transcription PCR primers

ADRB2 F: GCCTGTGCTGATCTGGTCAT.

ADRB2 R: AATGGAAGTCCAAAACTCGCA.

CKM F: CACCCCAAGTTCGAGGAGAT.

CKM R: AGCGTTGGACACGTCAAATA.

DUX4 F: GGCCCGGTGAGAGACTCCACAC.

DUX4 R: CCAGGAGATGTAACTCTAATCCAGGTTTGC.

DUX4 transgene F: TGACTGGATATGTTGTGTTTTAC.

DUX4 transgene R: CAACCCCGGATCCTTAGTG.

MBD3L2 F: GCGTTCACCTCTTTTCCAAG.

MBD3L2 R: GCCATGTGGATTTCTCGTTT.

MYOG F: GCCAGACTATCCCCTTCCTC.

MYOG R: GAGGCCGCGTTATGATAAAA.

RPL27 F: GCAAGAAGAAGATCGCCAAG.

RPL27 R: TCCAAGGGGATATCCACAGA.

RPL13A F: AACCTCCTCCTTTTCCAAGC.

RPL13A R: GCAGTACCTGTTTAGCCACGA.

TRIM43 F: ACCCATCACTGGACTGGTGT.

TRIM43 R: CACATCCTCAAAGAGCCTGA.

ZSCAN4 F: TGGAAATCAAGTGGCAAAAA.

ZSCAN4 R: CTGCATGTGGACGTGGAC.

#### siRNA target sequences

ADRB2 #1: GAGGGTAATAAACTTAGAATA (FlexiTube).

ADRB2 #2: CCAGGATAACCTCATCCGTAA (FlexiTube).

BRD2: GGTCTACCGGATTATCACA (Silencer Select).

BRD3: CGGCTGATGTTCTCGAATT (Silencer Select).

BRD4 #1: AGATTGAAATCGACTTTGA (Silencer Select).

BRD4 #2: TGAGCACAATCAAGTCTAA (Silencer Select).

Negative control #1: Silencer Select Negative Control #1.

Negative control #2: AATTCTCCGAACGTGTCACGT (FlexiTube).

## Results

### High-throughput screening approach to target DUX4 expression

Detection of DUX4 mRNA in FSHD muscle cells for drug screening purposes is challenging due to low transcript levels that reflect the fact that *DUX4* is expressed in only approximately 1 in 1000 FSHD myoblasts in culture [5], and that DUX4 mRNA is a target of nonsense-mediated decay [15] with a short half-life. Additionally complicating quantitative detection of the rare DUX4 mRNA are long sense and antisense transcripts that extend through the D4Z4 arrays on chromosome 4q and the nearly identical D4Z4 arrays on chromosome 10q, as well as an alternative splice form of DUX4 (DUX4s) that produces a truncated protein without transcriptional activity [5, 13, 21]. And although *DUX4* expression increases upon in vitro differentiation of proliferating FSHD myoblasts into multinucleated myotubes [26], the levels remain low and the dynamic variability is challenging for robust large-scale screening approaches.

Conversely, genes regulated by DUX4, most of which are undetectable in normal muscle but readily measurable in FSHD myoblasts and myotubes, provide abundant and stable RNAs that are sensitive and highly specific markers of *DUX4* expression [13, 27]. Further, reporter constructs derived from promoters of DUX4-responsive genes provide an additional readout of *DUX4* expression [13]. Thus, these target genes and derived reporters serve as excellent indicators of DUX4 mRNA and protein for use in high-throughput screens [28]. We carried out two such screens, differing primarily in the content of the compound libraries with minor distinctions in outcome measures, using FSHD muscle cells differentiated in culture to identify molecules that decrease DUX4 target gene induction.

### Screening identifies BET bromodomain inhibitors as blocking *DUX4* expression

In the first screen, we compiled several compound libraries that target epigenetic modifier proteins, representing about 100 modulators of known epigenetic “writers,” “readers,” and “erasers.” The identities of all the compounds in our final collection are included in Additional file 3: Table S2. We employed 54-2 FSHD1 muscle cells transiently transfected with a luciferase reporter containing the promoter of the DUX4 target gene *ZSCAN4* or a control reporter in which three of the four DUX4 binding sites present in the *ZSCAN4* promoter were mutated (Additional file 4: Figure S2A) [13]. Luciferase activity was detected when using the *ZSCAN4* reporters in 54-2 FSHD1 but not 54-6 control (non-FSHD) cells, and was dependent on intact DUX4 binding sites (Additional file 4: Figure S2B). The library compounds were screened at an initial concentration of 10 μM, or for some well-defined chemical probes at concentrations relevant for cell-based assays, for their ability to modulate the induction of the *ZSCAN4* promoter in differentiating 54-2 FSHD1 myotubes. A co-transfected internal control plasmid that expressed *Renilla* luciferase was used to monitor general compound toxicities as well as non-specific transcriptional effects. Variability in the luminescence assay precluded achievement of acceptable Z-scores for single-well compound screening. Therefore, compounds were screened in replicates of eight and visual inspection was included as a qualitative criterion for determining toxicity. In order to prioritize compounds for follow-up study, we ranked the average of the 8 replicate values and selected those molecules that inhibited *ZSCAN4* reporter expression > 50% (after normalization to internal control *Renilla* luciferase activity), had < 25% effect independently on *Renilla* luciferase activity, and showed no obvious cytotoxicity (evident morphological change or loss of cell adhesion) upon visual examination.

**Fig. 1:**
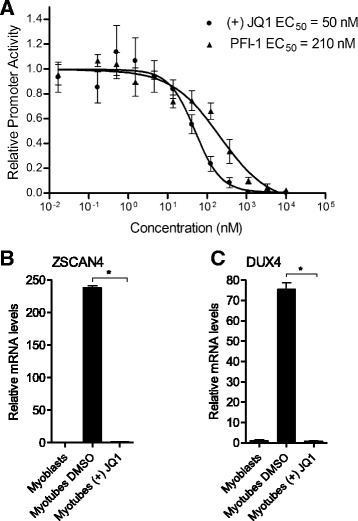
BETi block DUX4-mediated gene expression. **a** The BETi (+)-JQ1 and PFI-1 block *ZSCAN4* luciferase reporter activity in differentiating 54-2 FSHD1 myotubes with EC_50_s of 50 and 210 nM, respectively. **b**–**c** Treatment with 500 nM of the BETi (+)-JQ1 prevents induction of endogenous ZSCAN4 (**b**) and DUX4 (**c**) mRNA as 54-2 FSHD1 cells differentiate from myoblasts to 6-day-old myotubes. Relative mRNA levels for each gene were normalized to that in undifferentiated myoblasts, which was set to 1. *Error bars* indicate the standard deviation from the mean of three biological replicates. *p* values were calculated using a two-tailed, two-sample Student’s *t* test assuming unequal variance. *p < 0.05

This screening process identified four bromodomain-inhibiting compounds (Table 1): CBP/p300 bromodomain inhibitor SGC-CBP30, pan-bromodomain inhibitor bromosporine, and BET bromodomain inhibitors (BETi) (+)-JQ1 and PFI-1. Dilution curves revealed that (+)-JQ1 and PFI-1 completely prevented activation of the *ZSCAN4* promoter at high doses (> 1 μM) and resulted in half maximal effective concentrations (EC_50_s) consistent with the reported cellular potencies for each BETi (Fig. 1a) [29, 30].

**Table 1:** Hits from the epigenetic modifier screen

Compound	% inhibition of *ZSCAN4* reporter	% inhibition of *Renilla* luciferase	Mechanism
(+)-JQ1	99 (1 μM^a^)	27 (1 μM^a^)	BET bromodomain inhibitor
PFI-1	89 (1 μM^a^)	0 (1 μM^a^)	BET bromodomain inhibitor
SGC-CBP30	75 (1 μM^a^)	24 (1 μM^a^)	CBP/p300 bromodomain inhibitor
Bromosporine	92 (2 μM^a^)	9 (2 μM^a^)	Pan-bromodomain inhibitor

^a^Concentration of compound used in the assay

The luciferase readout in this screen was designed to reflect the expression of DUX4, which in turn modulates the *ZSCAN4* promoter. To determine whether BETi as a class block induction of *DUX4*, we measured DUX4 and DUX4 target gene mRNA levels after compound treatment in 54-2 FSHD1 and MB200 FSHD2 proliferating myoblast and differentiating myotube cell cultures using quantitative reverse transcription PCR﻿ (RT-qPCR). The BETi probe compound (+)-JQ1, the clinical candidate I-BET151, the Phase II clinical molecule I-BET762, and the Phase III clinical compound RVX-208 prevented the activation of *DUX4* and DUX4 target genes such as *ZSCAN4* and tripartite motif containing 43 (*TRIM43*) under all conditions tested (Fig. 1b–c **,** Additional file 5: Figure S3). These results demonstrate that BETi block *DUX4* expression in a manner independent of muscle differentiation in both FSHD1 and FSHD2 cells, suggesting that BETi act at a point in the transcriptional control of *DUX4* common to both genetic defects.

To further validate that BETi function primarily by inhibiting the expression of *DUX4* as opposed to affecting DUX4’s ability to induce target genes, we ectopically expressed DUX4 in 54-6 control (non-FSHD) myoblasts, added BETi at concentrations up to 20-fold higher than needed to block *DUX4* expression in FHSD myoblasts, and measured endogenous levels of the DUX4 target gene *MBD3L2*. BETi, even at high concentrations, did not block the ability of DUX4 to transactivate *MBD3L2* (Additional File 6: Figure S4). These results further support a mechanism by which BETi block *DUX4* expression, not activity, and substantiate the use of DUX4 target genes to monitor BETi activity.

### BET inhibition results in sustained suppression of *DUX4* expression that is mediated by lysine deacetylation but not DNA methylation

**Fig. 2:**
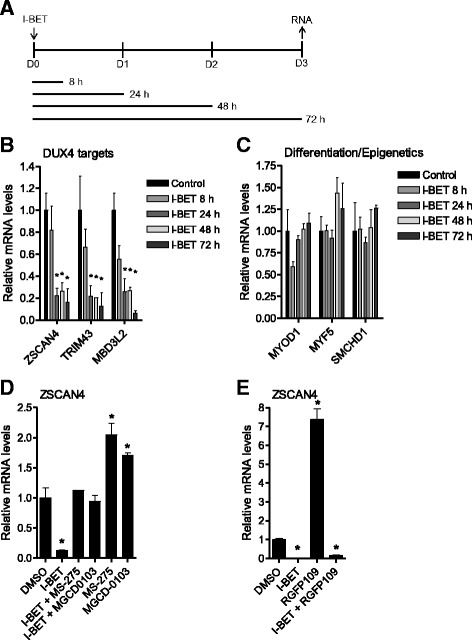
BETi have a memory effect on *DUX4* that is mediated by HDACs. **a**–**c** BETi cause sustained *DUX4* repression. **a** Experimental timeline. Subconfluent MB200 FSHD2 myoblasts were treated with 1 μM of the BETi I-BET762 (I-BET) on day 0 (D0) for 8, 24, 48, or 72 h and gene expression analyzed on day 3 (D3). **b** DUX4 target gene mRNA levels after treatment as in **a**. **c** Expression of the myoblast lineage genes *MYOD1* and *MYF5* and the epigenetic modifier *SMCHD1* after treatment as in **a**. **d**–**e** HDACi block the BETi-mediated memory effect. **d** Levels of the DUX4 target gene *ZSCAN4* in MB200 FSHD2 myoblasts that were treated with the indicated compounds (DMSO control, 1 μM I-BET762, 2.5 μM MS-275, 2.5 μM MGCD0103) for 24 h and then cultured in fresh media for an additional 48 h before harvest. **e** *ZSCAN4* expression after 72 h of continuous exposure to the indicated compounds (DMSO control, 1 μM I-BET762, 2 μM RGFP109) in MB200 FSHD2 myoblasts. *Error bars* indicate the standard deviation from the mean of three biological replicates. *p* values were calculated using a one-way analysis of variance with Dunnett’s post test. *p < 0.05

We determined that treating FSHD1 and FSHD2 myoblasts with BETi resulted in decreased DUX4 target gene expression that manifested over the course of several days, with maximal inhibition apparent after 48–72 h. To establish the time of drug exposure required to achieve the maximal response on DUX4 target gene expression, MB200 FSHD2 myoblasts were treated with 1 μM I-BET762 for various amounts of time up to 72 h (Fig. 2a). For exposures shorter than 72 h, drug-containing media was removed, the cells were rinsed before replacing fresh media without drug, and incubation continued until the 72-h time point. A 24-h pulse of I-BET762 resulted in sustained inhibition of the DUX4 targets *ZSCAN4*, *TRIM43*, and *MBD3L2*, while an 8-h exposure was less effective. In contrast, mRNAs for the myoblast lineage markers myogenic differentiation 1 (*MYOD1*) and myogenic factor 5 (*MYF5*) and the epigenetic modifier structural maintenance of chromosomes flexible hinge domain containing 1 (*SMCHD1*), the gene most commonly mutated in FSHD2 [11], were not affected (Fig. 2b-c). These data reveal a perdurance of BETi-mediated *DUX4* repression lasting up to 48 h after drug removal, which we refer to as the BETi-mediated memory effect.

Class I histone deacetylases (HDACs) have been previously implicated in suppressing *DUX4* expression [31]. Since BETi block binding of BET proteins to acetyl-lysine moieties [29, 30], we reasoned that the BETi-mediated memory effect on *DUX4* expression might occur by a mechanism in which HDACs remove the exposed acetyl groups during BET protein displacement. Therefore, inhibition of the appropriate HDACs during the period of BETi exposure should block the memory effect of sustained DUX4 suppression. Indeed, co-incubation of I-BET762 with the class I HDAC inhibitors MS-275 or MGCD0103 [32] for 24 h, followed by 48 h without I-BET762 or class I HDAC inhibitors, blocked the memory effect typically observed 48 h later (Fig. 2d). Additionally, a 24-h pulse of these class I HDAC inhibitors in the absence of I-BET762 stimulated DUX4 target gene expression at 72 h (Fig. 2d). These data are consistent with a dynamic role for class I HDACs in normally suppressing *DUX4* expression and in mediating the extended *DUX4* repression by BETi that lasts at least 48 h after drug removal. Similarly, continuous exposure of MB200 FSHD2 myoblasts to the highly selective class I HDAC inhibitor RGFP109 [33] for 72 h resulted in an increase in DUX4 target expression (Fig. 2e). However, this induction was completely blocked when I-BET762 was included during the entire 72-h assay. These data suggest that increased expression of *DUX4* during continuous inhibition of HDAC activity is associated with lysine acetylation and, since this increase is sensitive to BET inhibition, the recruitment of BET proteins for the activation of *DUX4* transcription.

*DUX4* de-repression in FSHD muscle coincides with a decrease in DNA methylation along the D4Z4 arrays [11, 34–36]. To determine if BETi-mediated suppression of *DUX4* expression was associated with re-establishment of DNA methylation at the D4Z4 repeats, we propagated MB200 FSHD2 myoblasts for 3 weeks in the continuous presence of low dose (+)-JQ1. These conditions maintained a 95% suppression of DUX4 targets that did not recover as long as compound was present (Additional file 7: Figure S5A). DNA methylation was measured along two regions of the D4Z4 repeat comprising 19 total CpG sites by bisulfite sequencing. Average methylation was significantly lower in FSHD2 cells than in control myoblasts, as expected, and continuous (+)-JQ1 exposure did not increase DNA methylation at the D4Z4 repeat in FSHD2 muscle (Additional file 7: Figure S5B-D). These data suggest that BETi block *DUX4* expression independent of DNA methylation and that maintaining decreased *DUX4* levels does not feedback to re-establish normal DNA methylation patterns at D4Z4 repeats.

Withdrawing (+)-JQ1 from MB200 FSHD2 cultures after 3 weeks of continuous treatment resulted in a slow recovery of DUX4 target gene expression, with ZSCAN4 mRNA reaching 20% of initial levels after 96 h of growth in drug-free media (Additional file 7: Figure S5E). This is consistent with earlier results (Fig. 2) and suggests a low rate of re-initiation of *DUX4* expression after BETi withdrawal.

### BRD4 mediates the activity of BET bromodomain inhibitors on DUX4 expression

The majority of disclosed BETi suppress the activity of all four mammalian BET proteins (BRD2, BRD3, BRD4, and bromodomain testis associated (BRDT)) [29]. We therefore examined the expression of BET genes in 54-6 control (non-FSHD) and 54-2 FSHD1 myoblasts and myotubes. Although *BRD2*, *BRD3*, and *BRD4* were robustly expressed (cycle times of 21–25) in undifferentiated myoblasts, *BRDT* was almost undetectable (cycle times > 40) (Additional file 8: Figure S6A-C). *BRD2*, *BRD3*, and *BRD4* were also mildly induced by differentiation in both control and FSHD1 myotubes, whereas *BRDT* was weakly induced only in FSHD1 myotubes, suggesting it is a DUX4 target gene. Our published chromatin immunoprecipitation-sequencing (ChIP-seq) and RNA-sequencing (RNA-seq) datasets generated from DUX4-expressing muscle cells [13, 37] suggest that the *BRDT* promoter is bound by DUX4, but show that very few full-length *BRDT* transcripts are present (Additional file 8: Figure S6D). Together, these data indicate that only BRD2-4 are candidates for mediating *DUX4* expression that is sensitive to BETi.

**Fig. 3:**
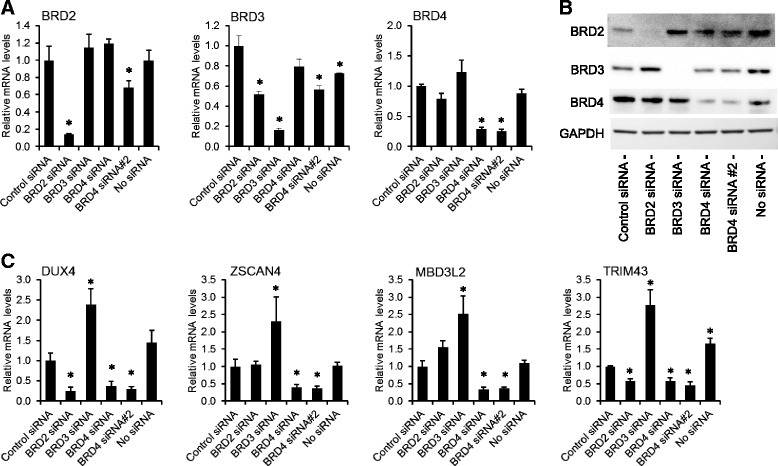
BRD4 depletion inhibits *DUX4* expression. **a**–**c** Expression of BET genes (**a**), BET proteins (**b**), and *DUX4* and DUX4 targets (**c**) after *BRD2*, *BRD3*, or *BRD4* siRNA knockdown in 54-2 FSHD1 myoblasts. *Error bars* indicate the standard deviation from the mean of three biological replicates. GAPDH serves as a loading control. *p* values were calculated using a one-way analysis of variance with Dunnett’s post test. *p < 0.05

We next performed siRNA knockdown experiments in 54-2 FSHD1 myoblasts to determine the necessity of each BET family member in mediating *DUX4* expression. We confirmed that siRNAs targeting *BRD2*, *BRD3*, and *BRD4* effectively depleted the relevant mRNA and protein (Fig. 3a–b). To assess the effect on *DUX4* expression, DUX4 and three DUX4 target gene mRNAs were quantified by RT-qPCR. *DUX4, ZSCAN4*, *MBD3L2*, and *TRIM43* were all selectively inhibited by BRD4 siRNAs, whereas *BRD2* knockdown had inconsistent effects (decreasing *DUX4* and *TRIM43* levels while having no impact on *ZSCAN4* or *MBD3L2*), and *BRD3* depletion resulted in increases in all four genes (Fig. 3c). Overall, these results demonstrate that *DUX4* expression (and thus expression of its downstream targets) is facilitated largely by BRD4 (and possibly by BRD2) in FSHD1 myoblasts, and indicate that these two BET proteins likely mediate the activity of BETi on *DUX4*.

### Second screen identifies beta-2 adrenergic receptor agonists as repressors of *DUX4* expression

For the second screen of the larger Pharmakon 1600 library, we developed an outcome measure based on the expression of the endogenous DUX4 target gene *MBD3L2* [13] using RT-qPCR, which introduced fewer variables into the screen than our initial transfected DUX4 reporter approach. Notably, the four hits from the epigenetic modifier library luciferase-based screen were all active in this second screen (Additional file 2: Figure S1). Although the second screen used an MB200 FSHD2 cell line and PCR detection of an endogenous DUX4 target gene rather than an FSHD1 line and luminescence measurement of a transfected DUX4 reporter, the major difference from the first screen was the library used. The Pharmakon library contains 1600 compounds that have reached clinical evaluation in the USA or internationally and includes advanced clinical compounds with known mechanisms of action. The compounds in this library are included in Additional file 9: Table S3. The library was initially screened at a concentration of 5 μM (see Methods for a detailed description of assay execution and performance). Drugs that reduced *MBD3L2* expression by > 65% were considered preliminary candidates and further subjected to 5-point dilution curves between 60 and 5000 nM to determine EC_50_s. To eliminate molecules that reduced *MBD3L2* expression due to toxicity or non-specific inhibition of muscle differentiation, cultures were scored on cytotoxicity and myotube formation by visual inspection, and *MBD3L2* levels were compared to those of the differentiation marker myosin heavy chain (*MYH2*). Candidates were excluded when visual inspection indicated overt cytotoxicity (loss of adherent cells) and/or myotube formation was < 50% of normal.

This screening process resulted in 52 compounds that selectively reduced *MBD3L2* expression (Table 2). Adrenergic receptor agonists comprised 25% of these 52 compounds, and 46% of these specifically target the beta-2 receptor (Table 2). These data indicate that adrenergic receptor activation, and in particular beta-2 adrenergic receptor activation, results in decreased *MBD3L2* expression during MB200 FSHD2 myotube differentiation. As a first validation step, selected adrenergic agonists were purchased as dry powders to ensure integrity and retested for their ability to modulate expression of *MBD3L2* (Additional file 10: Table S4). These data confirmed the potent activity of many beta-2 selective adrenergic agonists for decreasing *MBD3L2* in FSHD2 myotubes.

**Table 2:** Screening hits from the Pharmakon 1600 library

Drug	*MBD3L2* EC_50_ (nanomolar)	*MYH2* EC_50_ (nanomolar)	Mechanism
Clenbuterol hydrochloride	< 60	> 5000	Beta-2 adrenergic agonist
Tulobuterol hydrochloride	< 60	> 5000	Beta-2 adrenergic agonist
Albuterol	< 60	< 60	Beta-2 adrenergic agonist
Fenoterol hydrobromide	< 60	< 60^e^	Beta-2 adrenergic agonist
Fluticasone	< 60	< 60^d^	Glucocorticoid
Nylidrin (Buphenine isoxsuprine)	< 60	< 60^c^	Beta-2 adrenergic agonist
Sirolimus	< 60	< 60	Immunosuppressant
Terbutaline hemisulfate	< 60	< 60^c^	Beta-2 adrenergic agonist
Vinblastine	< 60^b^	< 60	Antineoplastic, spindle poison
Dequalinium	70	5000	Anti-infective, antineoplastic
Bisoctrizole	100^c^	> 5000	Sunscreen
Isoetharine	100	5000	Beta-1 and beta-2 adrenergic agonist
Penicillamine	180^c^	> 5000	Chelating agent
Benzethonium chloride	190	400	Anti-infective
Cetylpyridinium chloride	200	300	Anti-infective
Epinephrine bitartrate	200	4000	Alpha and beta-adrenergic agonist
Phenylephrine	200	4000	Alpha-1 adrenergic agonist
Piromidic acid	< 500^a^	> 5000	Antibacterial
Acenocoumarol	500^b^	> 5000	Anticoagulant
Atorvastatin	500^c^	> 5000	HmG-CoA reductase inhibitor
Cresopirine	500	> 5000	Anti-inflammatory, fever reducer
Dicoumarol	500^b^	> 5000	Anticoagulant
Thiostrepton	500	700^f^	Antibacterial
Thimerosal	500		Anti-infective, organomercury
Ethylnorepinephrine	600	5000	Alpha and beta adrenergic agonist
Fluvoxamine	1000	4000	SSRI, antidepressant
Clozapine	1000	2000	Antipsychotic
Adrenalone hydrochloride	1000	1500	Alpha-adrenergic agonist
Butamben	1000^f^	1000	Local anesthetic
Thonzonium	1000	1000	Mucolytic antibacterial
Mebendazole	1000^f^		Anthelmintic
Puromycin	1000^f^		Antibacterial, antineoplastic
Colforsin	1500^d^	> 5000	Adenylyl cyclase activator
Dimercaprol	1500	3000	Chelating agent
Benzalkonium	1500		Anti-infective
Norepinephrine	1600	4000	Alpha and beta adrenergic agonist
Polymyxin B	1700	3500	Antibacterial
Oxedrine (synephrine)	2000	5000	Alpha and beta adrenergic agonist and antagonist
Nonoxynol-9	2000		Spermicide
Papaverine	2500	10,000	Vasodilator
Pentamidine	3000		Antiprotozoal
Triamcinolone	3000		Corticosteroid
Dichlorephen	3500		Anthelmintic
Ebselen	4000		Antioxidant, anti-inflammatory
Minocycline	5000	10,000	Antibacterial
Inosine	5000	> 5000	Nucleoside
Sulbentine	5000	> 5000	Antifungal
Artesunate	5000		Anti-malarial
Broxaldine	5000		Antifungal
Broxyquinoline	5000		Anti-infectant
Meclocycline	5000		Antibacterial
Sennoside A	5000		Cathartic/laxative

^a^Maximum 50% inhibition

^b^Maximum 60% inhibition

^c^Maximum 65% inhibition

^d^Maximum 70% inhibition

^e^Maximum 85% inhibition

^f^Toxicity

**Fig. 4:**
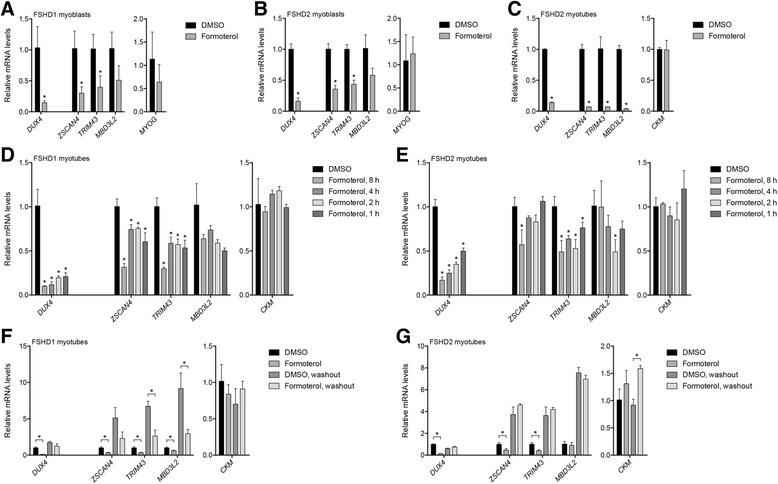
Beta-2 agonists inhibit DUX4-mediated gene expression. **a**–**g** Expression of *DUX4*, DUX4 target genes, and the myogenic markers *MYOG* or *CKM* in MB073 FSHD1 (**a**, **d**, **f**) or MB200 FSHD2 (**b**, **c**, **e**, **g**) myoblasts (**a**–**b**) or myotubes (**c**–**g**). Myoblasts were treated with 1 nM formoterol or DMSO vehicle control for 17 h. Myotubes were differentiated for 48 h with 1 nM formoterol or DMSO added during the final 24 h (**c**); the final 8, 4, 2, or 1 h (**d**–**e**); or the final 8 h after which compound was washed out and cells harvested 16 h later (**f**–**g**). *Error bars* indicate the standard deviation from the mean of three biological replicates. *p* values were calculated using a two-tailed, two-sample Student’s *t* test assuming unequal variance. *p < 0.05

To further validate whether beta-2 adrenergic agonists diminish *MBD3L2* through inhibiting *DUX4* expression, we analyzed mRNA for *DUX4* and several DUX4 target genes in MB073 FSHD1 and MB200 FSHD2 cell lines. Formoterol treatment of proliferating myoblasts and differentiating myotubes reduced *DUX4* expression and the expression of the DUX4 target genes *ZSCAN4*, *TRIM43*, and *MBD3L2* (Fig. 4a–c). These effects occurred independent of any impact to myogenesis, which was monitored by visually inspecting cell cultures and measuring levels of the myogenic genes myogenin (*MYOG*) and creatine kinase, M-type (*CKM*), without enhancing DUX4-mediated cell death (Additional file 11: Figure S7), and without affecting DUX4 transcriptional activity (Additional file 6: Figure S4). Beta-2 agonist-mediated reductions in *DUX4* expression were evident within 1 h of adding formoterol to the culture media (Fig. 4d–e), though *DUX4* repression was not sustained once the drug was removed (Fig. 4f–g).

### The beta-2 receptor mediates the activity of beta agonists on DUX4 expression

**Fig. 5:**
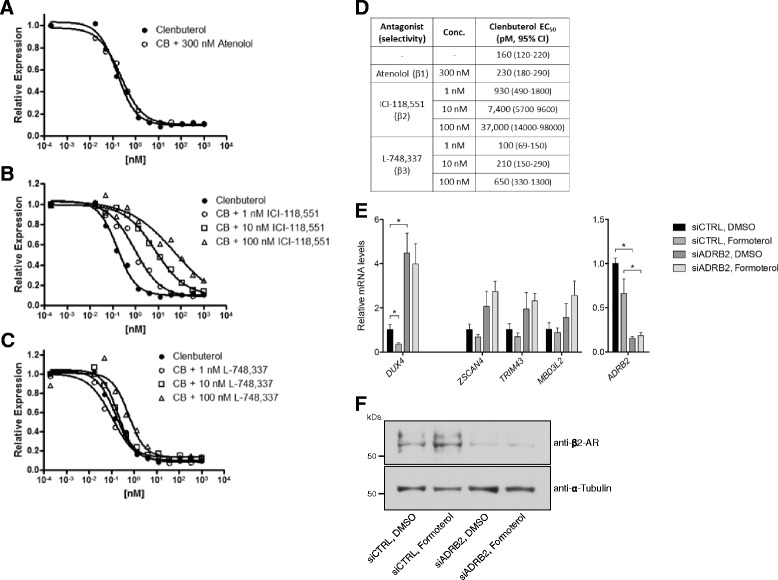
The beta-2 receptor mediates the activity of beta agonists on *DUX4* expression. **a**–**c** Concentration response curves for clenbuterol (CB) alone or in combination with the beta-1 selective antagonist atenolol (**a**), the beta-2 selective antagonist ICI-118,551 (**b**), or the beta-3 selective antagonist L-748,337 (**c**) on *MBD3L2* expression in MB200 FSHD2 myoblasts. **d** EC_50_s of clenbuterol alone or in combination with selective antagonists, indicated in picomolar (pM) concentrations with 95% confidence intervals (CI). **e** Expression of *DUX4*, DUX4 target genes, and the beta-2 receptor *ADRB2* after *ADRB2* siRNA knockdown in MB200 FSHD2 cells differentiated for 48 h with 1 nM formoterol or DMSO vehicle control added during the final 8 h. **f** Beta-2 adrenergic receptor (β2-AR) protein levels after *ADRB2* knockdown as described in **e**. *Error bars* indicate the standard deviation from the mean of three biological replicates. α-Tubulin serves as a loading control. *p* values were calculated using a two-tailed, two-sample Student’s *t* test assuming unequal variance. *p < 0.05

To determine the relative roles of the three beta receptors in mediating reductions in *DUX4* expression by beta agonists, we employed selective small molecule blockers of beta-1, beta-2, and beta-3 adrenergic receptors. Neither the beta-1 selective antagonist atenolol [38] nor the beta-3 selective antagonist L-748,337 [39] affected the clenbuterol EC_50_ at relevant concentrations (Fig. 5a, c, d). In contrast, the beta-2 selective antagonist ICI-118,551 [40, 41] shifted the concentration response curves for clenbuterol rightward in a dose-dependent manner, resulting in a statistically significant increase in the apparent EC_50_ for clenbuterol (Fig. 5b, d).

In addition, the conclusion that these compounds are acting as beta-2 agonists to inhibit *DUX4* levels was further supported by several other observations: (1) a beta-3 selective adrenergic agonist (CGP-12177A) did not inhibit *MBD3L2*; (2) a beta-2 agonist pro-drug (Bambuterol) was ineffective at blocking *MBD3L*2 expression, consistent with its requirement to be metabolized into the fully active form for action on the beta-2 receptor; and (3) phenylephrine, an alpha-1 selective adrenergic agonist, only weakly inhibited *MBD3L2*, consistent with its reported weak off-target activity on the beta-2 receptor [42] (Additional file 10: Table S4). Together, these pharmacologic data suggest a major role for beta-2 receptor activation in mediating the repressive effects on *DUX4* expression.

Depleting the beta-2 adrenergic receptor by transfecting MB200 FSHD2 cells with siRNAs targeting the beta-2 adrenergic receptor gene *ADRB2* genetically confirmed that beta agonists are reducing *DUX4* expression via the beta-2 adrenergic receptor (Fig. 5e–f). Upon siRNA knockdown of *ADRB2*, the ability of formoterol to decrease *DUX4* expression was abolished. In addition, *ADRB2* depletion in the absence of any formoterol treatment resulted in an increase in *DUX4* expression, suggesting that the beta-2 adrenergic receptor is important for controlling *DUX4* expression in the absence of synthetic ligands. Together with the pharmacologic data, these results demonstrate that beta-adrenergic agonist compounds act through the beta-2 adrenergic receptor to decrease *DUX4* gene expression.

### Pharmacologic modulation of the beta-2 receptor pathway

**Fig. 6:**
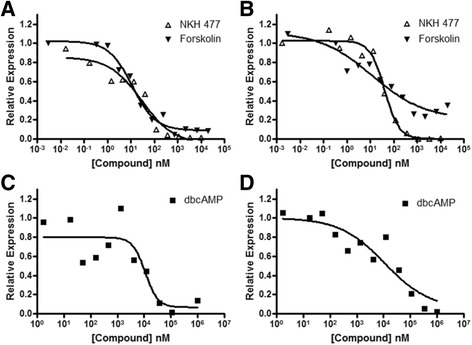
Elevated cAMP levels inhibit DUX4 target gene expression. **a**–**d** Expression of the DUX4 target gene *MBD3L2* in MB200 FSHD2 myoblasts (**a**, **c**) or myotubes (**b**, **d**) treated with the adenylyl cyclase activators NKH 477 or forskolin (**a**–**b**) or the cAMP analog dbcAMP (**c**–**d**) at the indicated concentrations. Data are normalized to untreated controls

Beta-2 agonist stimulation has long been known to promote muscle hypertrophy, in part by activating adenylyl cyclase to increase cellular cyclic adenosine monophosphate (cAMP) levels and stimulate protein kinase A (PKA) (for review see [43]). We used chemical modulators of these pathways to explore their effect on *DUX4* expression. Forskolin, and its more water-soluble analog NKH 477 (colforsin, a candidate inhibitor of *DUX4* in screen 2, see Table 2), are activators of adenylyl cyclase. When MB200 FSHD2 myoblasts or myotubes were exposed to forskolin or NKH 477, DUX4 target gene expression was reduced (Fig. 6a–b). Additionally, the stable cAMP analog dibutyryl cAMP (dbcAMP) reduced DUX4 target expression in MB200 FSHD2 myoblasts and myotubes (Fig. 6c–d). These data indicate that cellular cAMP levels are important for regulating *DUX4* expression and that beta-2 receptor agonists might suppress *DUX4* expression by modulating cAMP levels. In contrast, the PKA inhibitor H-89 provided only marginal recovery from the inhibition of DUX4 mRNA by formoterol treatment in differentiating MB073 FSHD1 or MB200 FSHD2 myotubes, and H-89 exposure in the absence of formoterol did not increase *DUX4* expression (Additional file 12: Figure S8). These results suggest that beta-2 agonists may not primarily utilize the PKA pathway to achieve inhibition of *DUX4* expression, and advance the possibility of PKA-independent regulation of the D4Z4 array.

## Discussion

DUX4 mis-expression in skeletal muscle is ultimately responsible for muscle degeneration in FSHD. Therefore, suppressing *DUX4* expression is a primary therapeutic approach for halting FSHD disease progression, and identifying drug targets for this purpose is a critical step. Much FSHD research to date has focused on genetic changes and the resultant loss of epigenetic silencing of *DUX4* within D4Z4 macrosatellite repeats in somatic tissue. However, a detailed mechanistic understanding of the process that results in transcriptional “bursts” of DUX4 in a subset of muscle cell nuclei [5, 18] is still lacking. Understanding this process is likely to produce drug targets with the potential to address the underlying cause of FSHD and the multiple mechanisms that contribute to disease pathology.

**Fig. 7:**
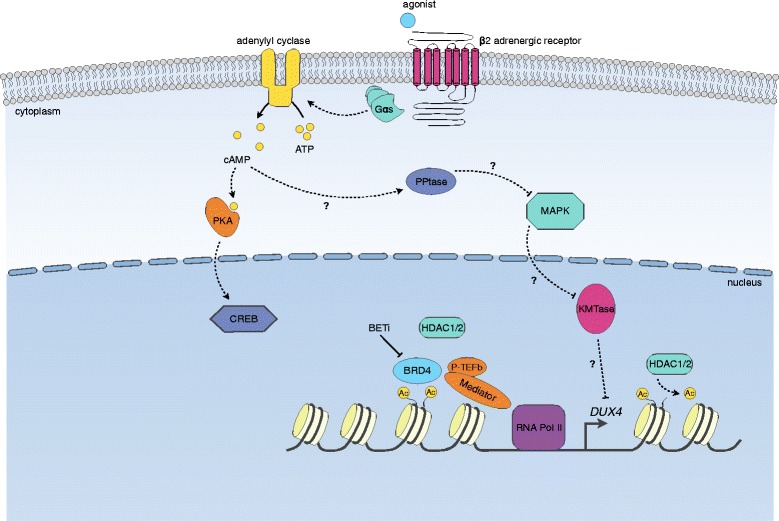
Model of *DUX4* regulation by BET proteins and beta-2 adrenergic signaling. BRD4 binds acetylated lysines at the D4Z4 array and recruits complexes such as P-TEFb and Mediator to facilitate transcriptional activation by RNA polymerase II. This is counteracted by the activity of HDAC1/2, which deacetylate lysines to inhibit gene induction. BRD4 binding to acetylated lysines may also shield these residues from HDAC1/2 activity. BETi prevent the binding of BRD4 to acetylated lysines and therefore block *DUX4* expression while allowing HDAC1/2 access to the exposed acetyl groups. Beta-2 agonist binding to the beta-2 adrenergic receptor results in Gs G protein-mediated activation of adenylyl cyclase, which catalyzes the formation of cAMP. Downstream effectors of cAMP include PKA-dependent and PKA-independent pathways. The inhibitory effect of beta-2 agonists on *DUX4* expression appears to be mostly mediated through PKA-independent pathways, possibly including the speculative one outlined here which is imagined to act through signaling molecules such as phosphatases (PPtases) and mitogen-activated protein kinases (MAPKs) to influence chromatin modifiers like lysine methyltransferases (KMTases) to impact transcription of the *DUX4* gene

As a complementary approach to previous studies on genetic and epigenetic changes, we have used chemical genetics to identify signaling pathways and epigenetic machinery that directly or indirectly influence *DUX4* expression in FSHD muscle. By screening libraries of chemical compounds with known mechanisms of action, we revealed unanticipated roles for BET proteins and beta-2 adrenergic receptor signaling in the processes controlling *DUX4* expression (Fig. 7). The novelty of these findings highlight the differences between the screens described here and those performed by others, who have used exogenous DUX4-mediated cytotoxicity in non-FSHD cells to identify broad inhibitors and mediators of cell death pathways [16, 22, 28], whereas our efforts uncovered specific regulators of the D4Z4 array that potentially provide more precise ways to disrupt FSHD pathogenesis. Importantly, it is not clear that merely blocking the most downstream consequence of *DUX4* expression—apoptosis—will prevent disease progression given the significant perturbations to fundamental cellular processes like RNA quality control, protein homeostasis, and the immune response that are present in DUX4-expressing muscle [13, 15, 18, 44]. However, it is also important to note that because *DUX4* expression has been reported in the testis, thymus, and possibly the skin [5, 6, 45], even a targeted therapeutic approach which envisions utilizing a small molecule to inhibit *DUX4* in FSHD muscle may also affect the normal function of some tissues, and potential side effects should be investigated.

Through our screens, we discovered that BETi prevent *DUX4* expression in FSHD muscle cells, uncovering a role for the proteins BRD4 (and possibly BRD2) in regulating the D4Z4 array. This is perhaps not unexpected, given that BET proteins have been widely shown to facilitate gene activation by recruiting transcriptional regulatory complexes to acetylated chromatin [46]. Indeed, the D4Z4 repeat contains acetylated histone H4 nucleosomes that are bound by epigenetic “readers” and “erasers” of this modification including histone deacetylase 1 (HDAC1), histone deacetylase 2 (HDAC2), and BRD4 itself [31, 47, 48] (Campbell et al. under review) (Fig. 7). There is intense interest by pharmaceutical companies in the development of potent and selective BETi as therapeutics for a myriad of diseases, and current trials seek to attenuate BET activity in settings as diverse as oncology, diabetes, and atherosclerosis; indeed, several studies have already combined current standard of care with BETi [49]. Given this level of interest and effort, BETi are candidates for future FSHD clinical trials. While the safety profile of BETi in cancer and other indications has been good, it is perhaps too soon to predict whether or not BET inhibition would offer a sufficient benefit to side effect profile for a chronic indication like FSHD. Potential complications may arise due to the wide expression pattern of BET proteins and their centrality to many cellular functions, including insulin production, cytokine gene transcription, T cell differentiation, adipogenesis, and repression of latent viruses like HIV [50].﻿ Therefore, choice of a BETi with properties tailored for FSHD will be critical going forward. These might include an optimized bromodomain binding profile for inhibiting *D﻿UX4* and appropriate pharmacokinetic properties for muscle exposure, among others.﻿

Our screening approach also enabled us to uncover a role for beta-2 adrenergic receptor signaling in modulating *DUX4* expression. This finding is unexpected based on our current understanding of D4Z4 regulation, and creates an important opportunity for future mechanistic studies to define the beta-2 adrenergic signaling pathways involved and uncover additional targets for drug development. Our experiments so far suggest that the canonical PKA signaling pathway downstream of the beta-2 receptor may not be mediating the effects on *DUX4*. Though restraint must be taken when interpreting these data as residual PKA activity may be enough to mediate *DUX4* suppression over the time course of our assay, it opens up the possibility that beta-2 agonists act on D4Z4 via alternative, PKA-independent pathways. It is interesting to speculate that these signaling cascades might impact chromatin modifiers that act on the D4Z4 array and therefore alter the epigenetic state of the locus (Fig. 7). Additionally, it is possible that alternative pathways will be more specific for D4Z4, and therefore have fewer potential side effects if they can be identified and targeted for therapeutic modulation. Interestingly, several clinical trials of the beta-2 adrenergic agonist albuterol have already been carried out in FSHD patients based on the drug’s known anabolic effects [51–54]. Although albuterol significantly increased muscle mass in these studies, this did not translate into functional improvements; however, better outcome measures to evaluate the effectiveness of such treatments for FSHD are needed before any potential therapy is disregarded. Such measures are being developed and tested, and include better FSHD-relevant functional measurements, self-reported measures of disease burden, and non-invasive imaging of disease progression [55–57].

Efforts to discover chemical inhibitors for use in the treatment of FSHD have lagged behind advances for other muscle disorders, likely due in part to the genetic complexity of this disease. However, the recent consensus of DUX4 as the causative factor in FSHD has provided a clear molecular target and allowed for the development of drug screens based on the activity of DUX4, such as measuring the expression of its target genes or reporters based on their promoters. Our screens used these two measures of DUX4 activity on different chemical libraries. The screens showed that endogenous activity of DUX4 in FSHD muscle cells can be used reliably to identify compounds that suppress *DUX4* expression. The libraries represented distinct classes of molecules, and it is encouraging that both of these small libraries yielded compounds that modulated *DUX4* expression and are already in clinical trials and/or use. This apparently high success rate provides hope that many additional candidate therapeutic compounds can be identified by a similar approach on larger and more complex libraries. The prospect of multiple initial therapeutic candidates makes it all the more imperative to not only actively prepare for clinical trials in FSHD, but to also anticipate that trials designed to efficiently compare compound efficacy without a full-scale clinical outcomes analysis will be important to prioritize which drugs to move to full clinical studies. As one example, multiple BETi compounds have been developed but it will take significant pre-clinical and focused clinical studies to determine which one(s), if any, to move forward into large-scale clinical trials, or how to assess BETi relative to other candidates, such as beta-2 adrenergic agonists. After decades of uncertainty regarding the pathophysiology of FSHD, these are refreshing new challenges.

## Conclusions

In summary, we identified small molecules that inhibit *DUX4* by screening several chemical libraries in FSHD patient-derived muscle cells while interrogating DUX4 target gene expression. These molecules—BET bromodomain inhibitors and beta-2 agonists—revealed an unexpected role for BET proteins and beta-2 adrenergic receptor signaling in the regulation of the D4Z4 array in somatic cells, and provide promising initial candidate classes of compounds for FSHD therapeutic development.

## Additional files

Additional file 1: Table S1. Characteristics of cell lines used in this study. (XLSX 31 kb)

Additional file 2: Figure S1. Primary screening data for the Pharmakon 1600 library. Quantitative PCR detection of DUX4 target gene *MBD3L2* is plotted for each library plate (88 compounds per plate). Data for each plate is normalized to no drug controls (*n* = 8), which were set to 1, as described in Methods. Plate means are indicated with horizontal lines and positive control data from each plate (I-BET762, *n* = 8) are collectively plotted in the rightmost column (Pos Controls). (PDF 126 kb)

Additional file 3: Table S2. Collection of compounds that target epigenetic modifier proteins. (XLSX 33 kb)

Additional file 4: Figure S2. The DUX4-responsive *ZSCAN4* reporter system used for screening epigenetic modifier compounds. (A) Schematic depicting the *ZSCAN4* promoter luciferase reporter vector (top) that includes four tandem DUX4 binding sites (D4BS), and a control reporter in which three of the four D4BS have been mutated (bottom). (B) Activity of the *ZSCAN4* reporter vectors in 54-2 FSHD1 and 54-6 control (non-FSHD) myoblasts and 6 day differentiated myotubes. Error bars indicate the standard deviation from the mean of three biological replicates. (PDF 66 kb)

Additional file 5: Figure S3. BETi block DUX4 target gene expression in FSHD myoblasts. (A) The expression level of ZSCAN4 mRNA after 72 h of treatment with the BETi (+)-JQ1, I-BET762, or I-BET151 in 54-2 FSHD1 myoblasts. (B) The BETi RVX-208 inhibits *ZSCAN4* and *TRIM43* expression in FSHD2 MB200 myoblasts treated for 72 h with EC_50_s of 350 nM and 280 nM, respectively. Error bars indicate the standard deviation from the mean of three biological replicates. (PDF 53 kb)

Additional file 6: Figure S4. BETi and beta-2 adrenergic agonists do not suppress the transcriptional activation function of DUX4. 54-6 control (non-FSHD) myoblasts that lack DUX4 were transfected with a DUX4 expression vector and DUX4 activity monitored by measuring mRNA levels of the endogenous DUX4 target gene *MBD3L2*. Compounds were added 5 h after transfection, a time at which there is little detectable *MBD3L2* expression, and mRNA levels assessed at 24 h after transfection. The level of *MBD3L2* at 24 h is expressed as ‘Fold Activation’ over the level present at 5 h, which was set to 1. Compounds were added in a 12 point, 3-fold dilution series to cover concentrations well above those required to inhibit *DUX4* expression. The data point farthest to the left for each of the compounds represents a DMSO (no drug) control. The approximate EC_50_s of the beta-2 adrenergic agonist clenbuterol (X) and the BETi I-BET762 (Y) for blocking *DUX4* expression in FSHD myoblasts are indicated on the x-axis. (PDF 31 kb)

Additional file 7: Figure S5. FSHD myoblasts grown continuously in low dose BETi maintain suppressed DUX4 target gene expression but do not re-establish D4Z4 repeat DNA methylation. (A) DUX4 target gene expression in MB200 FSHD2 myoblasts grown for three weeks in media containing DMSO or 100 nM (+)-JQ1 compared to gene expression in 54-6 control (non-FSHD) myoblasts. (B) Structure of the D4Z4 repeat unit showing regions analyzed by bisulfate sequencing in this study (ADS3747 and ADS1454) relative to the DUX4 open reading frame (ORF). ADS3747 spans positions 665-708 and ADS1454 spans positions 2230-2361 with respect to the start of the KpnI site. The locations of previously published methylation-sensitive restriction sites and regions analyzed by bisulfite sequencing (DR1, DR2 and DR3) are indicated. (C-D) Average percent methylation across 9 CpG sites within ADS3747 (C) or 10 CpG sites within ADS1454 (D) in 54-6 control (non-FSHD, Normal), 54-2 FSHD1, and MB200 FSHD2 myoblasts, as well as MB200 FSHD2 myoblasts grown for three weeks in media containing DMSO or 100 nM (+)-JQ1. (E) Slow recovery of DUX4 target gene expression after BETi withdrawal. MB200 FSHD2 myoblasts grown for 3 weeks in 100 nM (+)-JQ1 were split and seeded onto culture plates in the absence of drug at ~10% confluence to allow for continued growth. ZSCAN4 mRNA levels in untreated MB200 FSHD2 myoblasts (Control), MB200 FSHD2 myoblasts maintained continuously in drug (JQ1) and MB200 FSHD2 myoblasts grown for the indicated times after compound withdrawal are shown. Error bars in (A) and (E) indicate the standard deviation from the mean of three biological replicates. (PDF 122 kb)

Additional file 8: Figure S6. Characterization of BET gene expression. (A-C) 54-2 FSHD1 and 54-6 control (non-FSHD, Normal) myoblasts were induced to differentiate into myotubes and gene expression measured. Differentiation efficiency was determined by examining the early differentiation marker *MYOG* and the late differentiation marker *MYH2* (A). As expected, DUX4 targets were strongly induced upon differentiation of FSHD1 but not control myoblasts (B). The levels of BET family member (*BRD2*, *BRD3*, *BRD4*, *BRDT*) mRNAs are shown in (C). (D) Tracks showing ChIP-seq and RNA-seq reads mapped to the *BRDT* locus in DUX4-expressing muscle cells. Error bars indicate the standard deviation from the mean of three biological replicates. (PDF 117 kb)

Additional file 9: Table S3. Compounds present in the Pharmakon 1600 library. (XLSX 125 kb)

Additional file 10: Table S4. Retesting of adrenergic receptor agonists. (XLSX 38 kb)

Additional file 11: Figure S7. The effect of formoterol on DUX4-mediated cell death. MB135 control (non-FSHD) myoblasts that stably express a doxycycline-inducible DUX4 transgene [37] were used to test the effect of the beta-2 agonist formoterol on DUX4-mediated cell death. (A) Bright field images showing cell morphology after DUX4 expression at 24 h post doxycycline induction, with DMSO or 1 nM formoterol added during the last 16 h. (B) Cell counts from wells imaged in (A). (C) Expression of the DUX4 transgene and endogenous DUX4 target gene *MBD3L2* from cells treated as in (A). (D) Western blot showing expression of exogenous DUX4, endogenous MBD3L2, and endogenous Histone H3 as a loading control from cells treated as in (A). Error bars indicate the standard deviation from the mean of three biological replicates. (PDF 1068 kb)

Additional file 12: Figure S8. PKA inhibition does not prevent formoterol-mediated inhibition of *DUX4* expression. (**A-B**) Expression of *DUX4*, DUX4 target genes, and the myogenic marker *CKM* in MB073 FSHD1 (A) or MB200 FSHD2 (B) myotubes differentiated for 48 h and treated with 1 nM formoterol, 10 uM H89, or both compounds during the final 8 h of culturing. Error bars indicate the standard deviation from the mean of three biological replicates. *P*-values were calculated using a two-tailed, two-sample Student’s t-test assuming unequal variance. *, *p* < 0.05. (PDF 163 kb)

## Abbreviations

ADRB2: Adrenoceptor beta 2
BET: Bromodomain and extra-terminal
BETi: BET bromodomain inhibitor
BRD2: Bromodomain-containing protein 2
BRD3: Bromodomain-containing protein 3
BRD4: Bromodomain-containing protein 4
BRDT: Bromodomain testis associated
cAMP: Cyclic adenosine monophosphate
CDK4: Cyclin-dependent kinase 4
ChIP-seq: Chromatin immunoprecipitation-sequencing
CKM: Creatine kinase, M-type
dbcAMP: Dibutyryl cAMP
DMEM: Dulbecco’s Modified Eagle Medium
DMSO: Dimethyl sulfoxide
DUX4: Double homeobox 4
EC_50_: Half maximal effective concentration
FSHD: Facioscapulohumeral dystrophy
HDAC: Histone deacetylase
HDAC1: Histone deacetylase 1
HDAC2: Histone deacetylase 2
hTERT: Human telomerase reverse transcriptase
MBD3L2: Methyl-CpG binding domain protein 3 like 2
mRNA: Messenger RNA
MYF5: Myogenic factor 5
MYH2: Myosin heavy chain
MYOD1: Myogenic differentiation 1
MYOG: Myogenin
PCR: Polymerase chain reaction
PKA: Protein kinase A
RNA-seq: RNA-sequencing
RPL13A: Ribosomal protein L13a
RPL27: Ribosomal protein L27
RPL30: Ribosomal protein L30
RT-qPCR: Quantitative reverse transcription PCR
siRNA: Small interfering RNA
SMCHD1: Structural maintenance of chromosomes flexible hinge domain containing 1
TRIM43: Tripartite motif containing 43
ZSCAN4: Zinc finger and SCAN domain-containing 4
