# Supplementary figures and images for: BET bromodomain inhibitors and agonists of the beta-2 adrenergic receptor identified in screens for compounds that inhibit DUX4 expression in FSHD muscle cells
Source: Skelet Muscle. 2017 Sep 4;7:16. doi: 10.1186/s13395-017-0134-x (PMC5584331; doi:10.1186/s13395-017-0134-x)

Relative MBD3L2 Expression

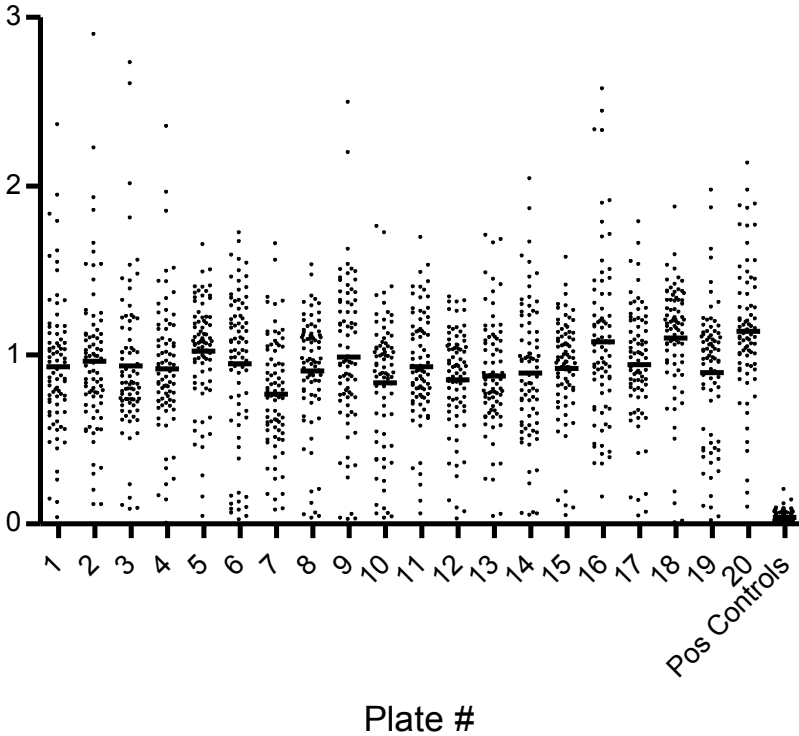

Supplement: Supplementary file 2 — Primary screening data for the Pharmakon 1600 library. Quantitative PCR detection of DUX4 target gene MBD3L2 is plotted for each library plate (88 compounds per plate). Data for each plate is normalized to no drug controls (n = 8), which were set to 1, as described in Methods. Plate means are indicated with horizontal lines and positive control data from each plate (I-BET762, n = 8) are collectively plotted in the rightmost column (Pos Controls). (PDF 126 kb) [file 13395_2017_134_MOESM2_ESM.pdf]

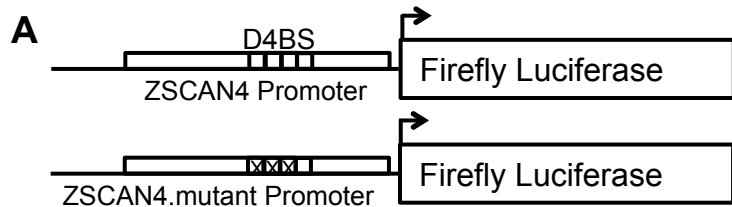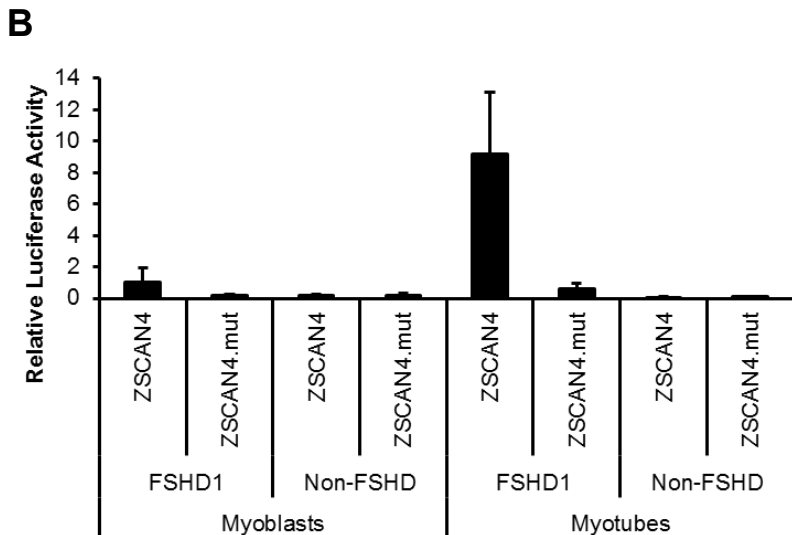

Supplement: Supplementary file 4 — The DUX4-responsive ZSCAN4 reporter system used for screening epigenetic modifier compounds. (A) Schematic depicting the ZSCAN4 promoter luciferase reporter vector (top) that includes four tandem DUX4 binding sites (D4BS), and a control reporter in which three of the four D4BS have been mutated (bottom). (B) Activity of the ZSCAN4 reporter vectors in 54-2 FSHD1 and 54-6 control (non-FSHD) myoblasts and 6 day differentiated myotubes. Error bars indicate the standard deviation from the mean of three biological replicates. (PDF 66 kb) [file 13395_2017_134_MOESM4_ESM.pdf]

**A**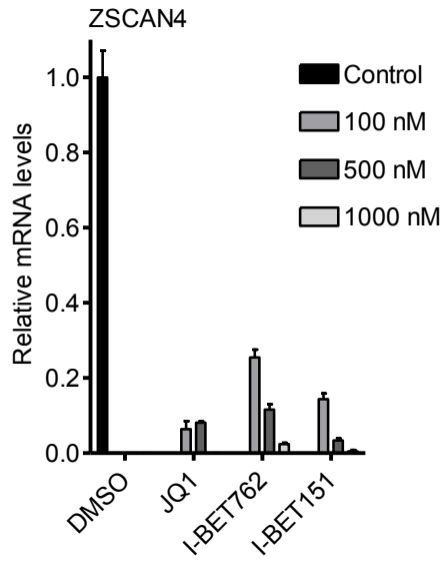**B**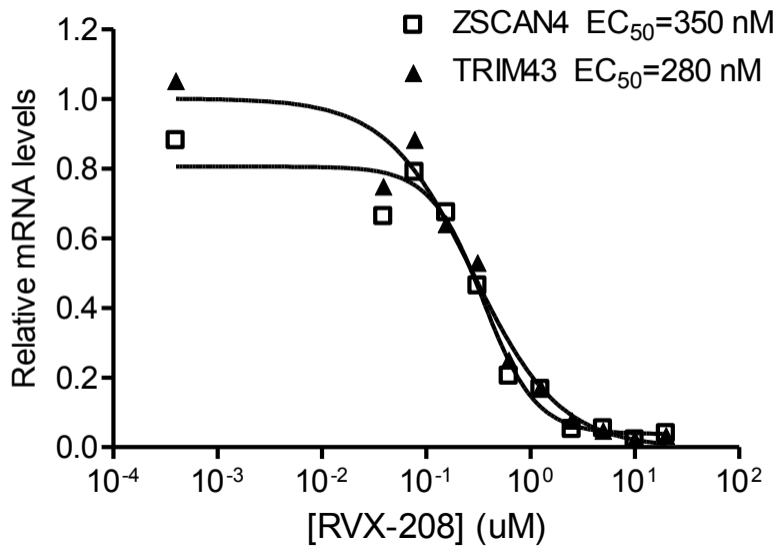

Supplement: Supplementary file 5 — BETi block DUX4 target gene expression in FSHD myoblasts. (A) The expression level of ZSCAN4 mRNA after 72 h of treatment with the BETi (+)-JQ1, I-BET762, or I-BET151 in 54-2 FSHD1 myoblasts. (B) The BETi RVX-208 inhibits ZSCAN4 and TRIM43 expression in FSHD2 MB200 myoblasts treated for 72 h with EC50s of 350 nM and 280 nM, respectively. Error bars indicate the standard deviation from the mean of three biological replicates. (PDF 53 kb) [file 13395_2017_134_MOESM5_ESM.pdf]

# MBD3L2

Fold Activation

● Clenbuterol  
▼ I-BET762

X Y

Drug Concentration (nM)

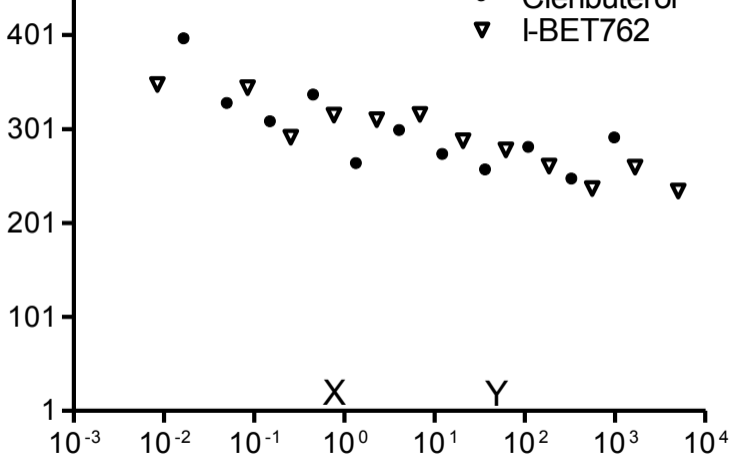

Supplement: Supplementary file 6 — BETi and beta-2 adrenergic agonists do not suppress the transcriptional activation function of DUX4. 54-6 control (non-FSHD) myoblasts that lack DUX4 were transfected with a DUX4 expression vector and DUX4 activity monitored by measuring mRNA levels of the endogenous DUX4 target gene MBD3L2. Compounds were added 5 h after transfection, a time at which there is little detectable MBD3L2 expression, and mRNA levels assessed at 24 h after transfection. The level of MBD3L2 at 24 h is expressed as ‘Fold Activation’ over the level present at 5 h, which was set to 1. Compounds were added in a 12 point, 3-fold dilution series to cover concentrations well above those required to inhibit DUX4 expression. The data point farthest to the left for each of the compounds represents a DMSO (no drug) control. The approximate EC50s of the beta-2 adrenergic agonist clenbuterol (X) and the BETi I-BET762 (Y) for blocking DUX4 expression in FSHD myoblasts are indicated on the x-axis. (PDF 31 kb) [file 13395_2017_134_MOESM6_ESM.pdf]

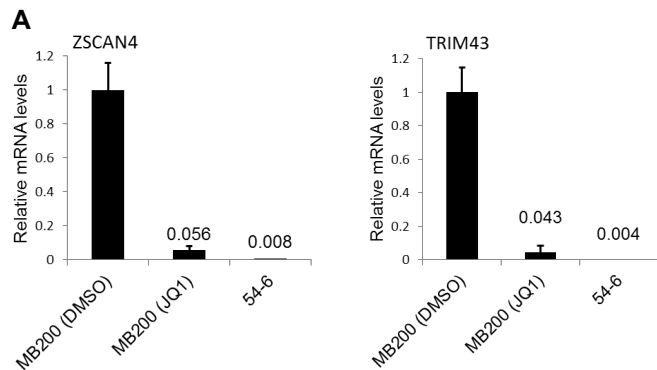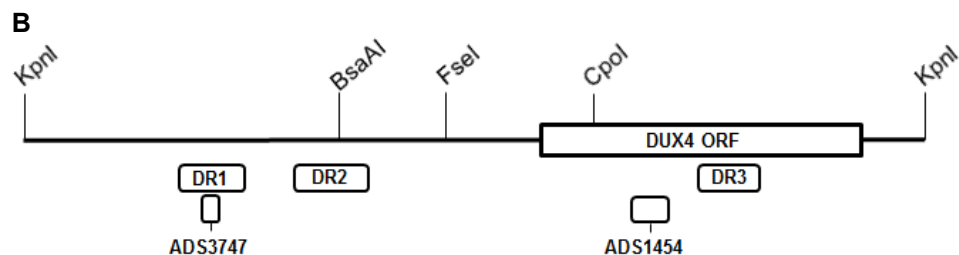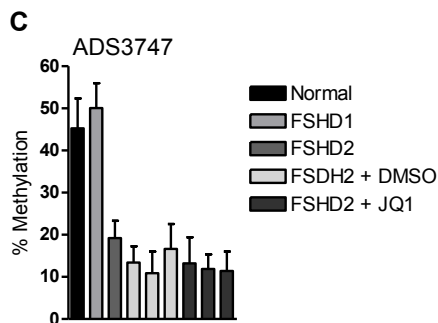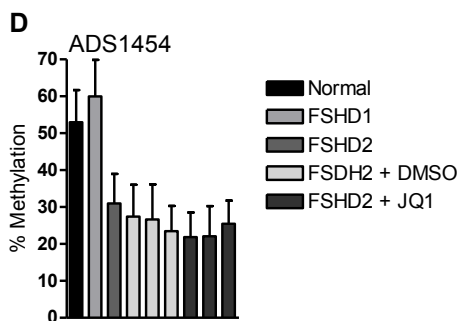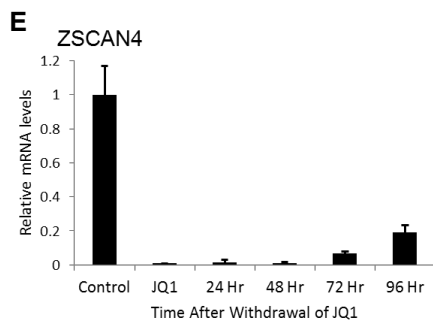

Supplement: Supplementary file 7 — FSHD myoblasts grown continuously in low dose BETi maintain suppressed DUX4 target gene expression but do not re-establish D4Z4 repeat DNA methylation. (A) DUX4 target gene expression in MB200 FSHD2 myoblasts grown for three weeks in media containing DMSO or 100 nM (+)-JQ1 compared to gene expression in 54-6 control (non-FSHD) myoblasts. (B) Structure of the D4Z4 repeat unit showing regions analyzed by bisulfate sequencing in this study (ADS3747 and ADS1454) relative to the DUX4 open reading frame (ORF). ADS3747 spans positions 665-708 and ADS1454 spans positions 2230-2361 with respect to the start of the KpnI site. The locations of previously published methylation-sensitive restriction sites and regions analyzed by bisulfite sequencing (DR1, DR2 and DR3) are indicated. (C-D) Average percent methylation across 9 CpG sites within ADS3747 (C) or 10 CpG sites within ADS1454 (D) in 54-6 control (non-FSHD, Normal), 54-2 FSHD1, and MB200 FSHD2 myoblasts, as well as MB200 FSHD2 myoblasts grown for three weeks in media containing DMSO or 100 nM (+)-JQ1. (E) Slow recovery of DUX4 target gene expression after BETi withdrawal. MB200 FSHD2 myoblasts grown for 3 weeks in 100 nM (+)-JQ1 were split and seeded onto culture plates in the absence of drug at ~10% confluence to allow for continued growth. ZSCAN4 mRNA levels in untreated MB200 FSHD2 myoblasts (Control), MB200 FSHD2 myoblasts maintained continuously in drug (JQ1) and MB200 FSHD2 myoblasts grown for the indicated times after compound withdrawal are shown. Error bars in (A) and (E) indicate the standard deviation from the mean of three biological replicates. (PDF 122 kb) [file 13395_2017_134_MOESM7_ESM.pdf]

**A**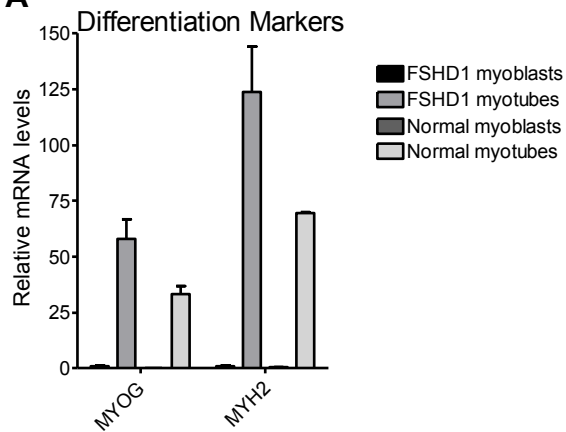**C**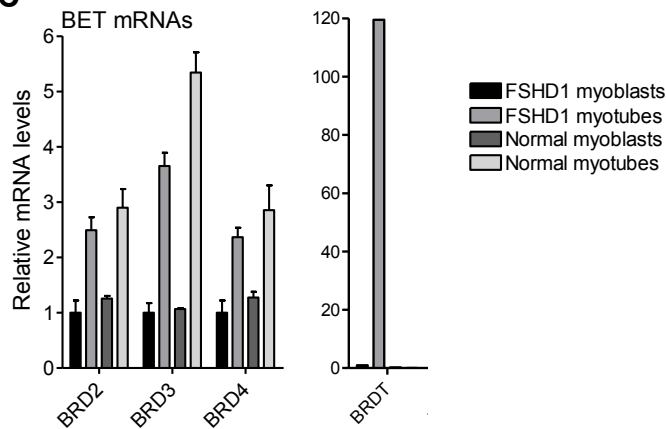**B**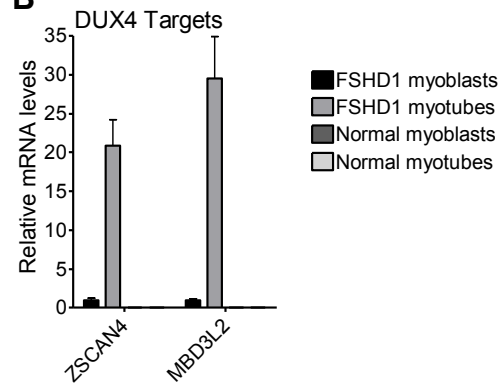**D**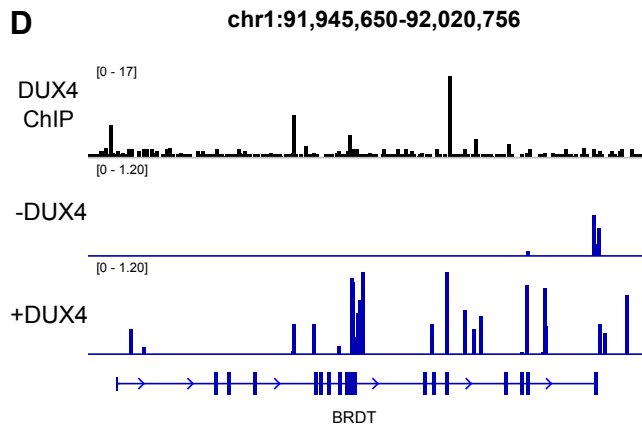

Supplement: Supplementary file 8 — Characterization of BET gene expression. (A-C) 54-2 FSHD1 and 54-6 control (non-FSHD, Normal) myoblasts were induced to differentiate into myotubes and gene expression measured. Differentiation efficiency was determined by examining the early differentiation marker MYOG and the late differentiation marker MYH2 (A). As expected, DUX4 targets were strongly induced upon differentiation of FSHD1 but not control myoblasts (B). The levels of BET family member (BRD2, BRD3, BRD4, BRDT) mRNAs are shown in (C). (D) Tracks showing ChIP-seq and RNA-seq reads mapped to the BRDT locus in DUX4-expressing muscle cells. Error bars indicate the standard deviation from the mean of three biological replicates. (PDF 117 kb) [file 13395_2017_134_MOESM8_ESM.pdf]

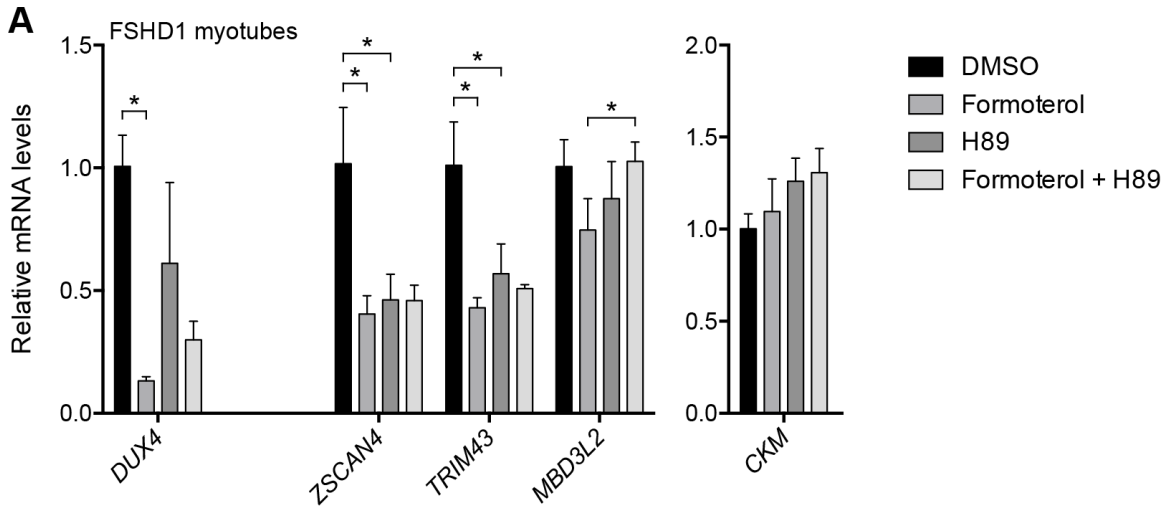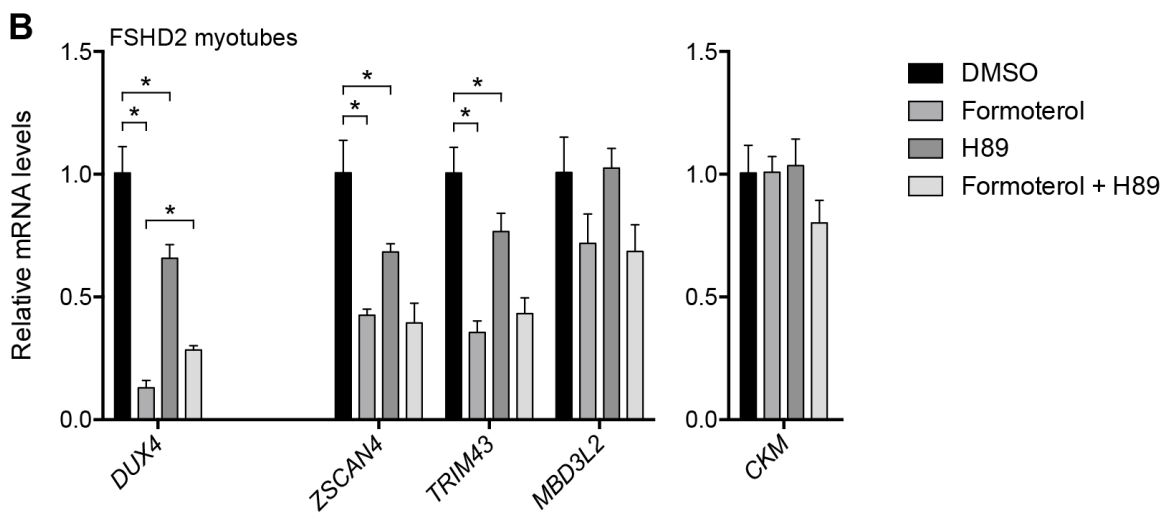

Supplement: Supplementary file 12 — PKA inhibition does not prevent formoterol-mediated inhibition of DUX4 expression. (A-B) Expression of DUX4, DUX4 target genes, and the myogenic marker CKM in MB073 FSHD1 (A) or MB200 FSHD2 (B) myotubes differentiated for 48 h and treated with 1 nM formoterol, 10 uM H89, or both compounds during the final 8 h of culturing. Error bars indicate the standard deviation from the mean of three biological replicates. P-values were calculated using a two-tailed, two-sample Student’s t-test assuming unequal variance. *, p < 0.05. (PDF 163 kb) [file 13395_2017_134_MOESM12_ESM.pdf]
