# Supplementary material for: BET bromodomain inhibitors and agonists of the beta-2 adrenergic receptor identified in screens for compounds that inhibit DUX4 expression in FSHD muscle cells
Source: Skelet Muscle. 2017 Sep 4;7:16. doi: 10.1186/s13395-017-0134-x (PMC5584331; doi:10.1186/s13395-017-0134-x)
Supplement: Supplementary file 11 — The effect of formoterol on DUX4-mediated cell death. MB135 control (non-FSHD) myoblasts that stably express a doxycycline-inducible DUX4 transgene [37] were used to test the effect of the beta-2 agonist formoterol on DUX4-mediated cell death. (A) Bright field images showing cell morphology after DUX4 expression at 24 h post doxycycline induction, with DMSO or 1 nM formoterol added during the last 16 h. (B) Cell counts from wells imaged in (A). (C) Expression of the DUX4 transgene and endogenous DUX4 target gene MBD3L2 from cells treated as in (A). (D) Western blot showing expression of exogenous DUX4, endogenous MBD3L2, and endogenous Histone H3 as a loading control from cells treated as in (A). Error bars indicate the standard deviation from the mean of three biological replicates. (PDF 1068 kb) [file 13395_2017_134_MOESM11_ESM.pdf]

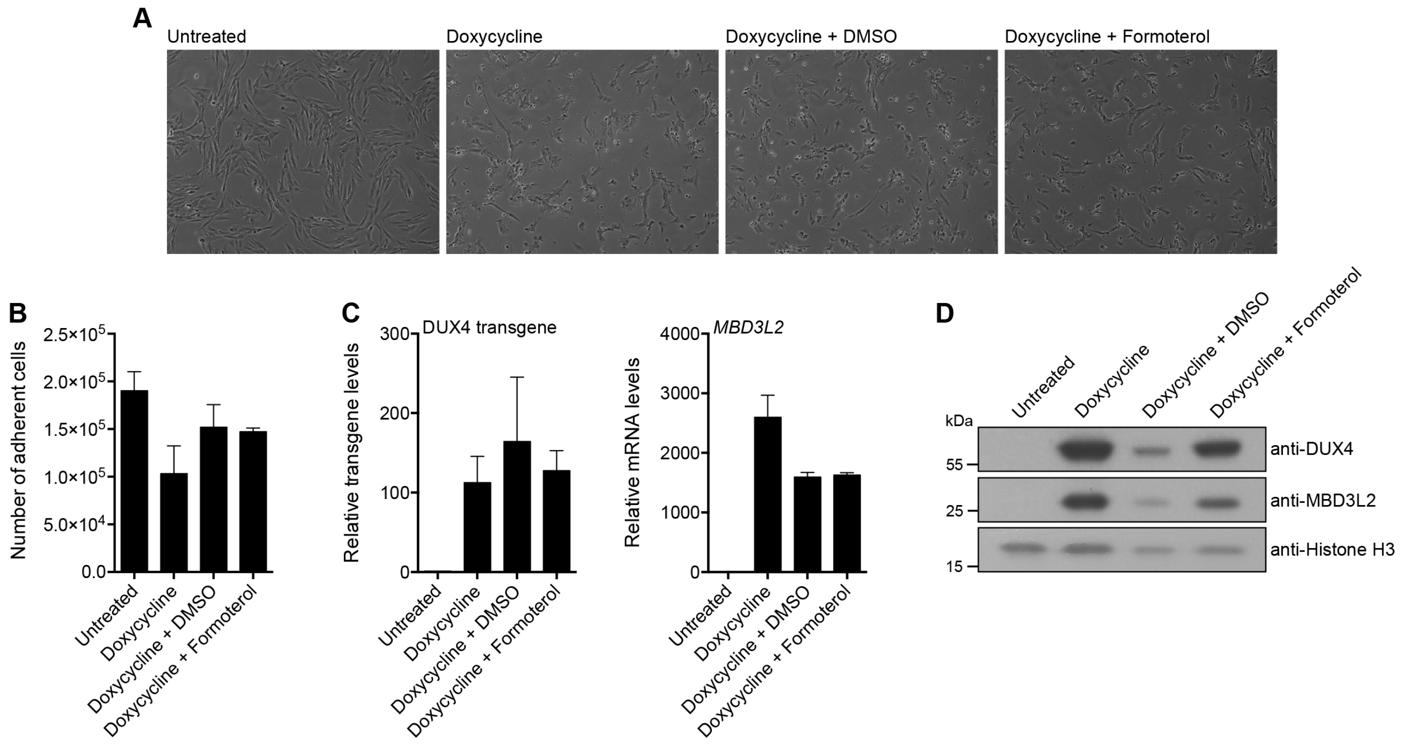
A
Untreated
Doxycycline
Doxycycline + DMSO
Doxycycline + Formoterol
B
Number of adherent cells
2.5×10^5
2.0×10^5
1.5×10^5
1.0×10^5
5.0×10^4
0.0
Untreated
Doxycycline
Doxycycline + DMSO
Doxycycline + Formoterol
C
DUX4 transgene
Relative transgene levels
300
200
100
0
MBD3L2
Relative mRNA levels
4000
3000
2000
1000
0
Untreated
Doxycycline
Doxycycline + DMSO
Doxycycline + Formoterol
D
Untreated
Doxycycline
Doxycycline + DMSO
Doxycycline + Formoterol
kDa
55
25
15
anti-DUX4
anti-MBD3L2
anti-Histone H3
